# Increase in bioethanol production from used office paper by *Saccharomyces cerevisiae *UFPEDA 1238

**DOI:** 10.1186/1753-6561-8-S4-P180

**Published:** 2014-10-01

**Authors:** David Lima, Ester Gouveia

**Affiliations:** 1 UFPE Universidade Federal de Pernambuco, PE, Brazil

## Background

Used paper is particularly attractive as feedstock for bioethanol production because it is readily available [1]. The aim this work was to hydrolyze used office paper with dilute sulfuric acid for bioethanol production by three industrial strains of *Saccharomyces cerevisiae*. Acid concentration, time of the hydrolyze and ratio solid:liquid were varying according to factorial design. Reaction time was not significant. Maximum total reducing sugar content was obtained with 5 % V/V acid concentration and 1:10 ratio solid-liquid. Higher ethanol production was obtained by UFPEDA 1238 in 24 h. Increase 7 to 47 % was obtained in ethanol production, when *S. cerevisiae *UFPEDA 1238 was used in relation to other two strains.

## Methodology

Used office paper of Department of Antibiotics from Federal University of Pernambuco, Brazil, was hydrolyzed with dilute sulfuric acid at 120 ºC and 1 atm (autoclave). The acid hydrolyzate was filtered through qualitative paper and used in the preparation of fermentation medium, after detoxification at room temperature by mixing with NaOH (4.5 pH). Acid concentration (1, 3 and 5 % V/V), time of the hydrolyze (60, 90 and 120 minutes) and ratio solid:liquid (1:50, 3:50 and 1:10) were varying according to factorial design. Three industrial strains of *Saccharomyces cerevisiae *(UFPEDA 1238, UFPEDA 1326 e UFPEDA 1337), were used in fermentations carried out at 34 ºC and 80 rpm. Samples were used for the determination of sugars, organic acids, furanic compounds and ethanol by high performance liquid chromatography on an Aminex HPX-87H^+ ^column at 60 ºC, 5 mM H_2_SO_4_, 0.6 mL/min and RI-detector [2]. The total reducing sugars (TRS) content of the acid hydrolyzed were measured using the 3,5-dinitrosalicylic acid reagent method [3].

## Results and conclusions

Increase in acid concentration and mass of paper increased the TRS. However, the reaction time was not significant. Maximum TRS content (28.40 g/L) was obtained with 5 % V/V acid concentration and 1:10 ratio solid-liquid. Starting this result hydrolyses at 10 % V/V acid sulfuric, with 10 g and 1 h in was carried out. Lower content of furfural and 5-hydroxymethyl furfural (HMF) were observed with 5 % V/V (A hydrolysate) than to 10 % V/V (B hydrolysate). In the acid hydrolyze, cellulose is hydrolyzed to glucose and hemicellulose is degraded to pentose and hexose. At high temperature and pressure, xylose and glucose are further degraded to furfural and HMF, respectively [4], which are inhibitors of the fermentation. Fermentations with A hydrolysate (5 % V/V; 0.9 M) were performed, using three strains of *S. cerevisiae*. Glucose was completely consumed in 24 h. Strain UFPEDA 1326 presented higher growth. On the other hand, higher ethanol production (1600 mg/L) was obtained by UFPEDA 1238 in 24 h. This study showed that ethanol production from used office paper is possible without the addition of cellulase enzyme.
